# Cryopreserved CD90+ cells obtained from mobilized peripheral blood in sheep: a new source of mesenchymal stem cells for preclinical applications

**DOI:** 10.1007/s10561-015-9526-5

**Published:** 2015-07-29

**Authors:** Carlos Landa-Solís, Julio Granados-Montiel, Anell Olivos-Meza, Carmina Ortega-Sánchez, Mónica Cruz-Lemini, Cecilia Hernández-Flores, María Eugenia Chang-González, Ricardo Gómez García, Brenda Olivos-Díaz, María Cristina Velasquillo-Martínez, Carlos Pineda, Clemente Ibarra

**Affiliations:** Department of Tissue Engineering, Cell Therapy and Regenerative Medicine, National Rehabilitation Institute, Av México-Xochimilco 289, Col. Arenal de Guadalupe, CP 14389 Mexico City, Mexico; Department of Fetal Medicine and Surgery, Women and Children’s Specialty Hospital of Queretaro, Queretaro, Mexico; National Institute of Pediatrics, Mexico City, Mexico; Research Direction, National Rehabilitation Institute, Av México-Xochimilco 289, Col. Arenal de Guadalupe, CP 14389 Mexico City, Mexico

**Keywords:** Mesenchymal stem cells, Mobilized peripheral blood, Cryopreserved CD90+ cells

## Abstract

Mobilized peripheral blood (MPB) bone marrow cells possess the potential to differentiate into a variety of mesenchymal tissue types and offer a source of easy access for obtaining stem cells for the development of experimental models with applications in tissue engineering. In the present work, we aimed to isolate by magnetic activated cell sorting CD90+ cells from MPB by means of the administration of Granulocyte-Colony Stimulating Factor and to evaluate cell proliferation capacity, after thawing of the in vitro culture of this population of mesenchymal stem cells (MSCs) in sheep. We obtained a median of 8.2 ± 0.6 million of CD90+ cells from the 20-mL MPB sample. After thawing, at day 15 under in vitro culture, the mean CD90+ cells determined by flow cytometry was 92.92 ± 1.29 % and cell duplication time determined by crystal violet staining was 47.59 h. This study describes for the first time the isolation, characterization, and post-in vitro culture thawing of CD90+ MSCs from mobilized peripheral blood in sheep. This population can be considered as a source of MSCs for experimental models in tissue engineering research.

## Introduction

There is currently a need to find sources of stem cells that offer high plasticity, permit their expansion in sufficient numbers for their use in experimental models in animals, where novel technologies can be developed for the repair of damaged tissues and that can safely be transferred to therapy in humans. This has encouraged investigators to experiment with mesenchymal stem cells (MSCs) mobilized in bone marrow (BM) to peripheral blood by means of treatment with Granulocyte-Colony Stimulating Factor (G-CSF) (Chao et al. 1993; Schmitz et al. 2005). This mechanism of action described for mobilizing MSCs to peripheral blood is based on the negative modulation of the surface molecule of Vascular Cell Adhesion Model-1 (VCAM-1) and inhibition of nestin in nestin-positive MSCs localized in the vascular compartment of the BM niche (Hopman and DiPersio 2014; Sousa et al. 2014), favoring its release from the niche and migration of these through the intramedullary cavity into peripheral circulation (Sahin and Buitenhuis 2012; Salvucci et al. 2012).

At the end of the past century, criteria were established for identification of MSCs in humans; adherence capacity to culture flasks under standard conditions of in vitro culture, expression of positive cell surface markers for CD73, CD90 and CD105 and negative for CD34 and CD45, and the differentiation capacity of at least three cellular lineages (osteoblastic, chondroblastic and adipogenic) (Dominici et al. 2006; Pittenger et al. 1999).

MSCs populations have been identified in mobilized peripheral blood (MPB) that has been characterized by CD105+, CD90+, CD73+, CD31−, CD34−, and CD45− surface markers (Rammal et al. 2013; Villa-Diaz et al. 2012). For the particular case of MPB-isolated MSCs, their plasticity has been demonstrated by differentiation to adipocytes, chondrocytes and osteoblasts (Jin et al. 2008; Tondreau et al. 2005).

Studies in which BM cells have been obtained from sheep have evaluated their potential as experimental models in the field of Orthopedics. They have been employed as CD29, CD44 and CD166 MSCs markers, in an attempt to establish the number of MSCs in sheep BM, obtaining positive cells to these antibodies (McCarty et al. 2009). However, in sheep MPB, cells that are positive for the CD90 MSCs marker have not been characterized, isolated or expanded in vitro.

Therefore, in the present study, we decided to isolate by means of magnetic pearls, the CD90+ cells of sheep MPS by means of the administration of G-CSF, and to evaluate the proliferation capacity of the in vitro culture of this MSCs population.

## Materials and methods

### Characteristics of the sheep

We employed four male Suffolk sheep weighing between 60 and 70 kg, who were stabled in an adequate area designed as an animal facility. Sheep were managed by establishing their individual clinical history, describing in detail state of health and body condition, upon their admittance into the animal facility. All animals received human care in compliance with the “Guide for the Care and Use of Laboratory Animals” published by the National Institutes of Health (NIH publication 85-23, 1985, cited 2011). Experimental studies were conducted in accordance with NOM-062-Z00-1999, the Animal Protection Law for the Federal District and the General Health Law Related to Health Research (2001).

### Bone marrow cell mobilization to peripheral blood in sheep

For mobilization of stem cells to peripheral blood, two sheep received three doses every 24 h of G-CSF (Filgrastim; Amgen, Thousand Oaks, CA, USA) at a dose of 10 μg/kg body weight, subcutaneously (sc). On day 4, they were administered 20 mL of MPB from the jugular vein mixed with heparin 100 IU/mL, also sc.

### Pre- and post-operative care given to sheep for MPB obtention

MPB obtention was performed with the sheep standing without sedation, because the procedure was minimally invasive and no discomfort in the animals was observed during or after it. For sampling, the first jugular groove area was shaved, antisepsis of the area was conducted with a solution of iodine and 70 % ethyl alcohol, threefold. A 16G needle was used for phlebotomy, taking into account the sample volume collected (20 mL). The sample was poured into 4 sterile (12 × 75 mm) tubes with heparin. All the tubes were gently shaken before sending them to the laboratory for processing. After drawing the MPB sample, the sheep returned to its yard where it was monitored by the animal facility staff until recovery.

### Isolation of CD90+ cells

The CD90+ cells contained in the peripheral blood of the sheep after mobilization with G-CSF were separated, first together with the mononuclear cells by means of the concentration gradient, in a laminar flow bell (Forma Scientific, Inc., Marietta, OH, USA). Each sample was placed in polypropylene tubes (50-mL capacity) for centrifugation. Subsequently, a 1:2 dilution with phosphate buffer solution (PBS) (Gibco Invitrogen, Grand Island, NY, USA) was carried out, with addition of antibiotics/antimycotics at 1 % (Penicillin 10,000 UI, Streptomycin 10,000 µg, and Amphotericin B 25 µg). We prepared 15 mL of Ficoll Paque (Amersham Biosciences, Piscataway, NJ, USA) in sterile, 50-mL polypropylene tubes (cat. CLS430829, Corning) and added 25 mL of the sample diluted in blood and PBS, with great care in order not to break surface tension, and to achieve a final volume of 40 mL. Later, this was centrifuged at 400*g* for 30 min. After centrifuging, a fraction of mononuclear cells was taken to initiate the CD90+ cell separation procedure, using the magnetic-activated cellular separation kit (Miltenyi cat. 130-042-303) with the anti-CD90 monoclonal antibody (Miltenyi cat. 130-096-253) coupled with magnetic particles through LS cell separation columns (Miltenyi cat. 130-096-253). From the total number of CD90+ cells obtained, we produced 1 × 10^5^ cell aliquots for characterization by Flow Cytometry (FC) and aliquots of 5 × 10^5^ cells/mL for cryopreservation in fetal bovine serum (Gibco cat. 10082139) supplemented with 10 % dimethyl sulfoxide (Sigma-Aldrich cat. D5879). Samples were then stored in liquid nitrogen at −196 °C.

### Characterization by flow cytometry

For the CD90+ MSC authentication, after isolation of the mononuclear cells, we set apart a 5 × 10^5^ cell aliquot in 1 mL for evaluating the presence of stem cell markers by means of FC. The marking procedure was as follows: once the cells had been separated from the Ficoll and washed with PBS, a portion of approximately 2.5 × 10^4^ cells are placed in polystyrene tubes [Falcon; Becton–Dickinson (BD)] with 10 µL of the antibody suspension and were left to incubate for 30 min at 4 °C. The monoclonal (directly conjugated) antibodies applied were: FITC-conjugated CD90 (50 μg/ml, mouse IgG1κ, cat. 555595), PE-conjugated CD14 (20 μg/ml, mouse IgG2bκ, cat. 340660), FITC-conjugated CD105 (5 μg/ml, mouse IgG1κ, cat. 561443), and PE-conjugated CD166 (20 μg/ml, mouse IgG1κ, cat. 559263) all from BD PharMigen™ (California, USA). The samples and or unlabelled controls were included for each antibody and used to set the gating on the flow cytometer. Data were acquired in a BD FACSCalibur flow cytometer and analyzed by CellQuest™ PRO software (Becton–Dickinson) with a mean of 20,000 events. This procedure was repeated each time that CD90+ cells were obtained from the sheep.

### Culture of the mesenchymal stem cells

After 2 months, cryopreserved CD90+ cells were thawed and cultured. We proceeded to expand for each research subject, a 5 × 10^3^ cell aliquot by triplicate in 2-dimensional (2D) culture in Dulbecco’s Modified Eagle’s Medium (DMEM; Gibco-Life Technologies, USA, cat. 11960-044), enriched with 10 % adult sheep serum (SBA; BIO-WEST, Inc. cat. S4190-100), with addition of antibiotics/antimycotics at 1 % (Gibco-Life Technologies). The cultures were maintained in an incubator at 37 °C with 5 % of CO_2_, in 6-well culture plates for a 15-day period until 90 % confluence was reached. Crystal violet-technique staining was performed at days 2, 4, 8, 11 and 15. The cells maintained in culture up to day 15 were marked with the previously described panel of antibodies and we proceeded to conduct their analysis by FC to establish immunophenotype.

### Characterization by immunofluorescence

CD90+ cells after 15 days of culture, were first passed to a mononuclear layer and fixed with 2 % paraformaldehyde for 20 min. Each sample was washed with 0.5 mL of PBS, followed by a solution of PBS/albumin 1 %/triton 0.3 % during 20 min to block unspecific binding sites. Subsequently, primary antibodies were incubated overnight at 4 °C at a concentration of 10:40 µL using the following antibodies: anti-CD14, (100 µg at 1 mg/ml, mouse IgG1κ, ABCAM cat. ab6083), anti-CD166 (100 µg at 1 mg/ml, mouse IgG, ABCAM cat. ab78649), anti-CD90 (100 µg at 1 mg/ml, mouse IgG1κ, ABCAM cat. ab225) and CD105 (200 µg/ml, rat IgG2aκ SANTA CRUZ cat. sc-71042). Afterwards, it was washed 2 times with PBS/triton 0.1 % and secondary antibodies anti-IgG-FITC (molecular probes, cat. 65-6111), anti-IgG1-FITC (ABCAM, cat. ab97239) and anti-IgG2a-FITC (ABCAM, cat. ab97244) were placed, coupled to a fluorophore. Control isotype antibodies were also used: mouse IgG1, kappa monoclonal-isotype control (ABCAM cat. ab170190); rabbit IgG, polyclonal-isotype control (ABCAM cat. ab171870) and mouse IgG2a, kappa monoclonal-isotype control (ABCAM cat. ab18415), at a concentration of 1:50 µL at 37 °C for 2 h. It was washed once more with PBS/triton 0.1 % to remove the excess secondary antibody. Finally, the slides were mounted with DAPI mounting medium Vectashield (Vector cat. H-1200). The images were captured in a pyramid microscope Carl Zeiss Axio system image Vision 4.8.2.

### Determination of cellular proliferation

Cell proliferation determination was carried out by means of crystal violet staining technique as previously described by Kueng et al. (1989). For this, we removed the culture medium and left the culture to air-dry. Immediately afterwards, the cells were fixed with glutaraldehyde 1.1 % (Sigma-Aldrich, cat. G5882) for 10 min, after which we removed the excess of fixative, washed the cells with distilled water and again left them to air-dry. We then proceeded to stain the cells with crystal violet dye at 0.1 % (Sigma-Aldrich) for 10 min, after which we removed the excess dye by means of washes with distilled water and left to air-dry. Finally, the dye was solubilized in acetic acid (Sigma-Aldrich) at 10 % under shaking for 20 min, and absorbance was measured at 620 nm in a DTX 800 spectrometer (Beckman–Coulter). Photographs of the stained cells in the wells were taken with a Zeiss-brand invertoscope with the AxioVision ver. 4.8.2 software program. To calculate duplication time of the cells under culture, we first calculated the growth rate utilizing the following formula: (median absorbance value obtained at day 15 minus absorbance value obtained at day 2) divided by (absorbance value obtained at day 2), multiplied by 100. Finally, the calculation of cellular duplication time was obtained by dividing the duplication constant (70) by the percentage value of the growth rate, with the result expressed in hours.

### In vitro differentiation of mobilized MSCs into mesenchymal tissues

In order to characterize MSCs obtained from MPB and demonstrate their functionality before and after cryopreservation, in vitro differentiation into mesenchymal tissues like osteoblasts, chondrocytes, and adipocytes was induced. Cells were cultured in six-well plates at a plating density of 1.6 × 10^3^ cells/cm^2^.

For osteogenic differentiation, a 70 % subconfluent culture of mobilized MSCs from passage P2 was used. Cells were incubated in osteogenic medium containing: DMEM, medium supplemented b-FGF (10 ng/mL), BMP-2 (10 ng/mL), β-glycerol phosphate (10 mM), ascorbic acid (50 μg/mL), and dexamethasone (1 × 10^−6^ M). After 15 days bone cell nodules were observed positive for alkaline phosphatase. For cartilage differentiation, the same cell confluency was used. Cultures were incubated for 15 days in chondrogenic medium, containing: DMEM, medium supplemented b-FGF (10 ng/mL), kartogenin (10 μM), and ascorbic acid (50 μg/mL). Media were changed every third day. For adipogenic differentiation, cells were incubated in medium containing: DMEM, medium supplemented b-FGF (10 ng/mL), dexamethasone (1 × 10^−9^ M) and insulin (1 μM). Cultures were incubated during 15 days and media were changed every third day.

### Cell layer and tissue staining

Cell cultures were stained for alkaline phosphatase, Alcian blue, and oil red O staining assays. Cartilage matrix deposition in cells cultured with chondrogenic medium before and after thaw was assessed by Alcian blue staining. Cell layers were stained with Alcian blue (1 % in 3 % acetic acid) for 30 min, washed three times for 2 min in 3 % acetic acid, rinsed once with water. To assess osteoblast differentiation, alkaline phosphatase staining was used. Cell layers were extracted in 2 mL of lysis buffer (100 mM Tris–HCl, pH 9.0, 200 mM NaCl, 0.2 % Nonidet P-40, 0.2 % Triton X-100, 1 mM MgSO_4_, 1 mM phenylmethylsulfonyl fluoride and 10 μg/mL aprotinin) by rotating plates for 30 min at 4 °C. Reaction mixtures, containing 50 μL of extract, 200 μL buffer, and 250 μL of phosphatase substrate (1 mg/mL in 20 % diethanolamine-HCl, pH 9.8), were incubated for 30 min at 37 °C. Oil red O staining assay was used to measure adipogenic differentiation. Medium was aspirated from plate dishes and cells were washed once in PBS. Culture layers were fixed in 4 % PFA for 15 min and washed twice with ddH_2_O for 2–5 min. Oil red O working solution to stain was applied and left for 15 min; cells were then washed three times with 1× PBS for 5 min. Finally, cells were rinsed with 50 % isopropanol once and 1× PBS once.

### Statistical analysis

All data are expressed as mean ± standard deviations (SD). Statistical analysis was performed with the STATISTICA StatSoft ver. 7 data analysis program. We compared all cell surface markers expressed in CD90+ cells isolated on the day of MPB sampling and on day 15 of them under culture. Comparison was performed by the Mann–Whitney *U* test. A statistically significant difference was considered when the value was *p* < 0.05.

## Results

We obtained a 20-mL sample of MPB from each sheep on day 4, after the first dose of G-CSF was administered, with a mean of 19.6 × 10^6^ ± 0.76 × 10^6^ mononuclear cells after separation of the concentration gradient with the Ficoll, and a mean of 8.2 × 10^6^ ± 0.58 × 10^6^ CD90+ cells isolated from mononuclear cells by means of magnetic pearls.

**Fig. 1:**
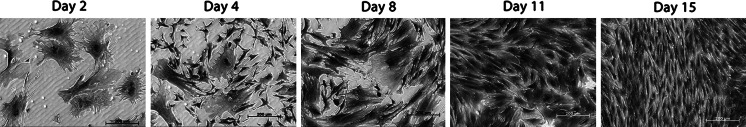
Morphology and proliferation potential by crystal violet staining at days 2, 4, 8, 11 and 15 of in vitro culture after thawing of mobilized peripheral blood (MPB)-isolated CD90+ cells in sheep

**Fig. 2:**
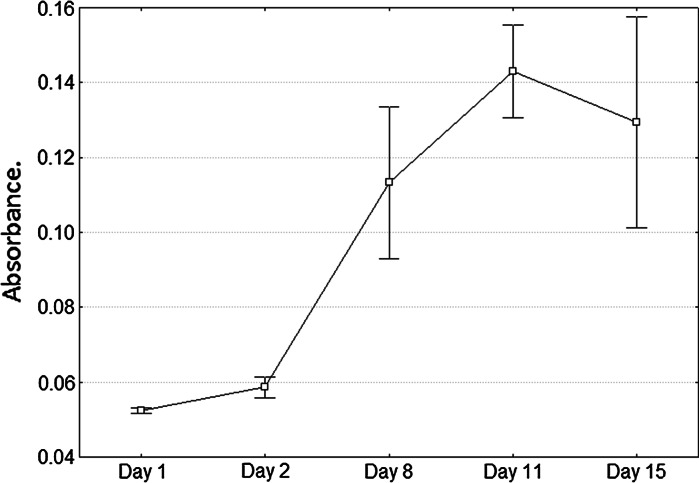
Proliferation in vitro of CD90+ cells isolated from quantified MPB cells after thawing, by crystal violet staining, through absorbance measurement at 620 nm

Crystal violet staining of CD90+ cells after thawing is shown in Fig. 1, with an established duplication time for MPB-isolated CD90+ cells of 47.59 h (Fig. 2).

**Fig. 3:**
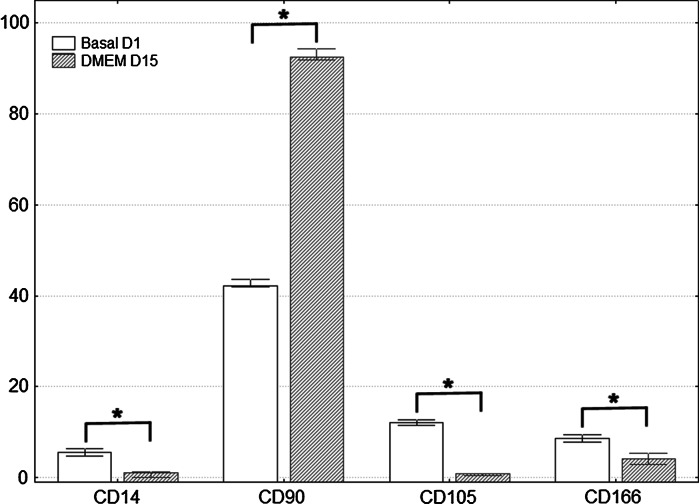
Comparison of immunophenotype determined by flow cytometry for the panel of CD14, CD90, CD105 and CD166 antibodies, from the day CD90+ cells were isolated from MPB (Basal D1) and day 15 of culture after thawing (DMEM D15). *p < 0.05 by comparison with Mann–Whitney *U* test

**Fig. 4:**
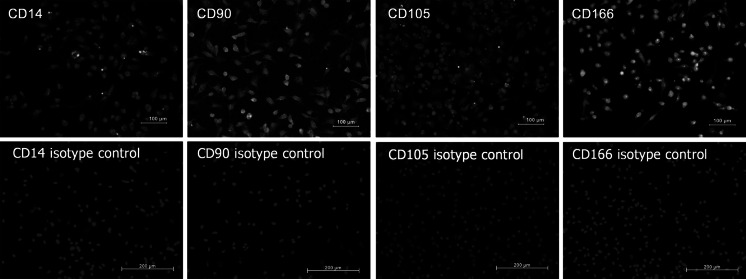
Immunofluorescence in CD90+ isolated cells from MPB after thawing at 15 days of monolayer culture, for superficial cell markers CD14, CD90, CD105, and CD166. Cell nuclei present as *blue* when stained with 4′ 6-diamidino-2-phenylindole [DAPI], and *green* with fluorescein isothiocyanate (FITC) fluorochrome-labeled antibodies. (Color figure online)

**Fig. 5:**
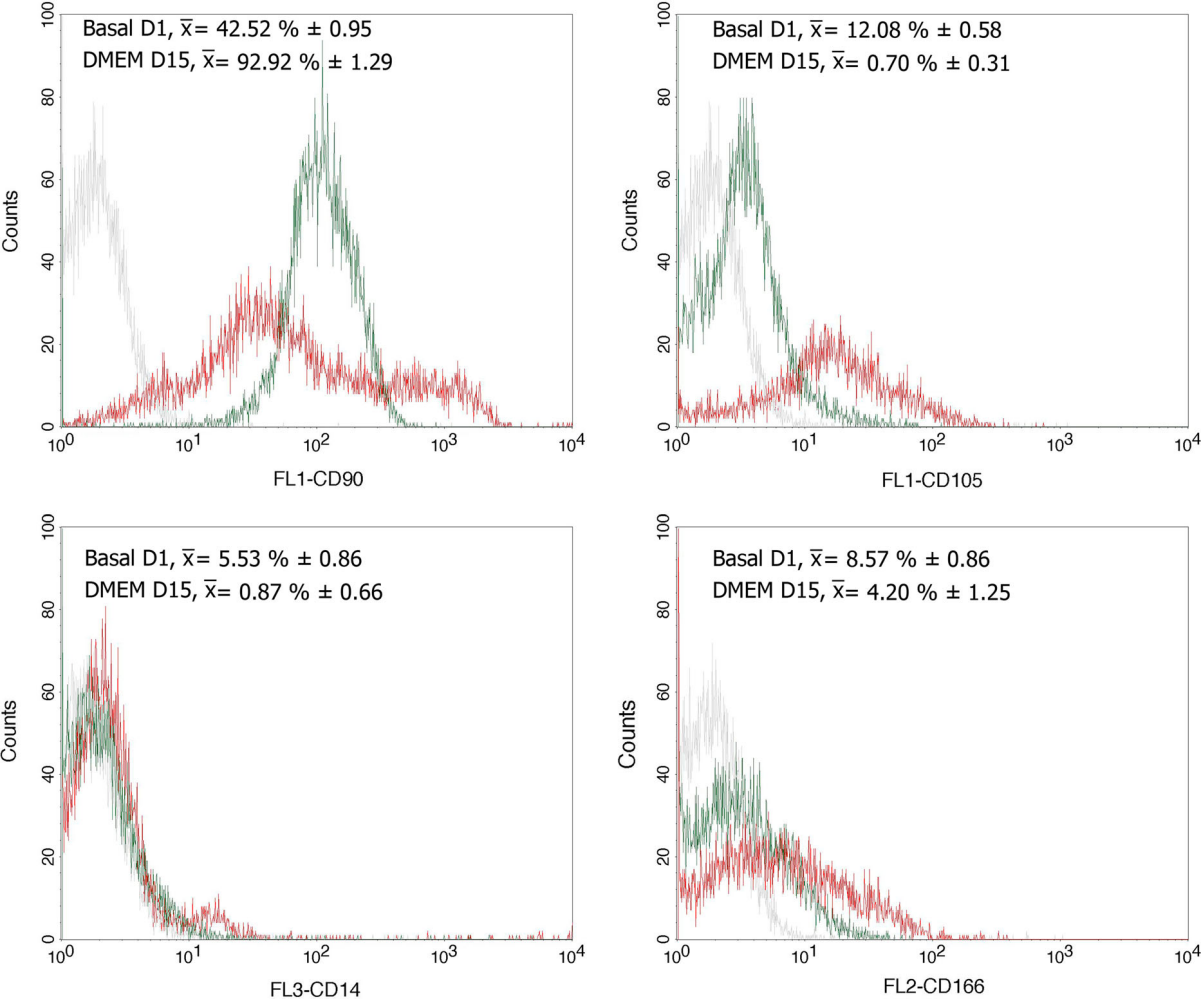
Immunophenotyping of CD90+ cells isolated from MPB. Data represent the percentage of positive cells for each fluorochrome that was conjugated with each of the monoclonal antibodies used, against the number of events detected. *Gray peak*, isotype control; *red peak*, basal CD90+ cells before cryopreservation (Basal D1); *green peak*, CD90+ cells after thawing in primary culture for 15 days (DMEM D15). (Color figure online)

**Fig. 6:**
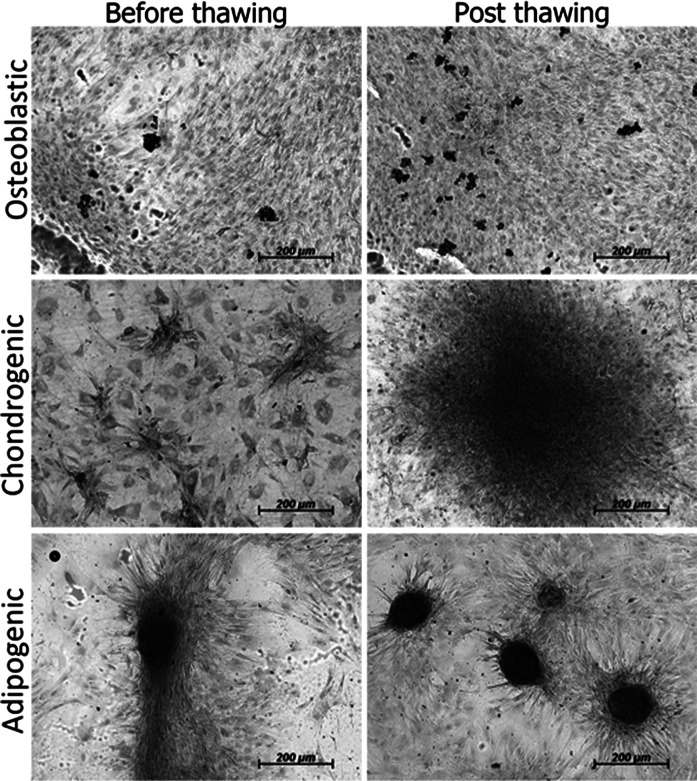
In vitro differentiation of mobilized MSCc into mesenchymal tissues. Positive cells for alkaline phosphatase (osteoblasts, *first row*), Alcian *blue* positive staining for cartilage differentiation (*middle row*) and oil *red* O for adipocyte differentiation (*last row*)

Immunophenotype for cells on the day CD90+ cells were isolated was as follows: CD14 5.53 ± 0.86 %; CD90 42.52 ± 0.95 %; CD105 12.08 ± 0.58 % and CD166 8.57 ± 0.86 %. At day 15 under culture, immunotypes were the following: CD14 0.87 ± 0.66 %; CD90 92.92 ± 1.29 %; CD105 0.70 ± 0.31 %, and CD166 4.20 ± 1.25 %. Comparisons with the Mann–Whitney *U* test among cell surface marker readings on the first day and on day 15, were statistically significant for the increase in the percentage of markers for CD90 mesenchymal cells, while the opposite occurred for the CD14 monocyte marker and for the markers of mesenchymal cells CD105 and CD166 (Fig. 3). Characterization by immunofluorescence of surface markers for CD14, CD90, CD105 and CD166 is shown in Fig. 4, where at 15 days of culture in vitro after thawing, the expression of these markers is retained in the cells. Figure 5 shows histograms for each of the markers overlayed with the control isotypes. In vitro differentiation of mobilized MSCs into mesenchymal tissues after 15 days showed differentiation towards 3 lineages: osteoblastic, chondrogenic and adipogenic, positive for their corresponding staining techniques (Fig. 6).

## Discussion

This study describes for the first time isolation and characterization in vitro of CD90+ MSCs from Mobilized peripheral blood (MPB) in sheep. Our results show isolation is viable for the population of CD90+ MSCs cells, as well as their expansion in vitro after thawing.

The sheep is an ideal model for biomedical research; it has been proposed as an animal model for a broad gamma of applications, such as tissue engineering, study of respiratory diseases, cardiomyopathies, neurological disorders, and prion diseases (Lyahyai et al. 2012). Studies have been conducted in the field of tissue engineering for MSCs obtained from BM in sheep (Lacitignola et al. 2014; Weber et al. 2011). However, MSCs isolation and characterization in MPB in sheep has been sparsely studied (Juthier et al. 2006), and the majority of what has been described in the literature has been focused on the obtaining and characterization of hematopoietic stem cells (Almeida-Porada et al. 2007; Porada et al. 2008). Isolation of MSCs in MPB had been previously reported in a variety of mammalian species including pigs (Harn et al. 2013), rabbits (Fu et al. 2014), dogs (Thomasson et al. 2003), mice (Lee et al. 2014), rats (Deng et al. 2014), and humans (Kang et al. 2014).

Due to the fact that collection of peripheral blood is a less invasive procedure than a BM biopsy, currently considered the best source of MSCs (Bieback et al. 2008), the mobilization of MSCs of BM to peripheral blood through the employment of G-CSF would represent a significant advantage for patients in the design of future clinical applications, such as autologous transplantation of chondrocytes (Ibarra et al. 2014). Our group, through characterization of a MSCs population (CD90+) in sheep, sought to establish the foundation for future projects of cellular differentiation to tissues of interest such as cartilage. As a result of this project, we standardized the separation techniques with MACS for CD90+ cells, its immunophenotype by FC, and duplication time and optimal conditions for CD90+ cell propagation under in vitro culture after 2 months of cryopreservation in liquid nitrogen at −196 °C. We believe this population of CD90+ cells can be considered a source of MSCs for experimental tissue-repair models in the future.

## Conclusion

Isolation and in vitro characterization of CD90+ MSCs from mobilized peripheral blood in sheep is feasible. Our results show isolation is viable for the population of CD90+ MSCs cells, as well as their expansion in vitro after thawing. This population of CD90+ cells could be considered a source of MSCs for experimental tissue-repair models, with considerable clinical applications in the near future.
